# RACIAL/ETHNIC DIFFERENCES IN SUBJECTIVE COGNITIVE DECLINE (SCD) AND SCD-INDUCED OUTCOMES

**DOI:** 10.1093/geroni/igad104.3552

**Published:** 2023-12-21

**Authors:** Moyosoreoluwa Jacobs, Peter Lichtenberg, Wassim Tarraf

**Affiliations:** Wayne State University, Detroit, Michigan, United States; Wayne State University, Detroit, Michigan, United States; Wayne State University, Detroit, Michigan, United States

## Abstract

Alzheimer’s disease and related dementias (ADRD) prevalence will double by 2060, with significant racial/ethnic (R/E) differences in rates. Understanding R/E differences in risk factors of subjective cognitive decline (SCD)—a precursor to ADRD—is critical for timely premorbid interventions to modify ADRD onset. We used 2015 CDC Behavioral Risk Factor Surveillance System data from non-Hispanic White (NHW), Black (NHB), and Hispanic individuals aged ≥45 years (n=142,657) to examine R/E differences in SCD Status and five SCD-induced functional limitations and healthcare outcomes (e.g., health provider communication). We used generalized linear modeling to assess the contributions of predisposing (e.g., age), enabling (e.g., insurance), and health need (e.g., chronic disease) factors to explaining R/E differences. NHWs were generally older and healthier, with higher educational attainment than NHBs and Hispanics. NHBs (13.5%) were slightly more likely to endorse SCD (11.5% overall). NHBs and Hispanics endorsing SCD were significantly more likely than NHWs to report SCD-induced functional limitations. R/E differences were minimally attenuated after adjusting for the above-mentioned factors. We found no R/E differences in getting access to help, or in communicating with healthcare providers. NHBs and Hispanics (vs. NHWs) endorsing SCD are more likely to also endorse functional limitations despite reporting equivalent access to help and communication with healthcare providers. Clinical cognitive impairment depends on functional status and as such NHBs and Hispanics are potentially more vulnerable to cognitive disease (e.g., ADRD) onset. More research is required to understand gaps in care and how interventions can help mitigate this differential risk.

